# Supplementing Exposure to Hypoxia with a Copper Depleted Diet Does Not Exacerbate Right Ventricular Remodeling in Mice

**DOI:** 10.1371/journal.pone.0092983

**Published:** 2014-04-15

**Authors:** Ella M. Poels, Nicole Bitsch, Jos M. Slenter, M. Eline Kooi, Chiel C. de Theije, Leon J. de Windt, Vanessa P. M. van Empel, Paula A. da Costa Martins

**Affiliations:** 1 Department of Cardiology, CARIM School for Cardiovascular Diseases, Faculty of Health, Medicine and Life Sciences, Maastricht University, Maastricht, The Netherlands; 2 Department of Radiology, CARIM School for Cardiovascular Diseases, Maastricht University Medical Centre, Maastricht, The Netherlands; 3 Department of Respiratory Medicine, NUTRIM School Nutrition, Toxicology and Metabolism, Maastricht University Medical Centre, Maastricht, The Netherlands; 4 Department of Cardiology, Heart Vessel Center, Maastricht University Medical Centre, Maastricht, The Netherlands; Vanderbilt University Medical Center, United States of America

## Abstract

**Background:**

Pulmonary hypertension and subsequent right ventricular (RV) failure are associated with high morbidity and mortality. Prognosis is determined by occurrence of RV failure. Currently, adequate treatment for RV failure is lacking. Further research into the molecular basis for the development of RV failure as well as the development of better murine models of RV failure are therefore imperative. We hypothesize that adding a low-copper diet to chronic hypoxia in mice reinforces their individual effect and that the combination of mild pulmonary vascular remodeling and capillary rarefaction, induces RV failure.

**Methods:**

Six week old mice were subjected to normoxia (N; 21% O_2_) or hypoxia (H; 10% O_2_) during a period of 8 weeks and received either a normal diet (Cu+) or a copper depleted diet (Cu-). Cardiac function was assessed by echocardiography and MRI analysis.

**Results and Conclusion:**

Here, we characterized a mouse model of chronic hypoxia combined with a copper depleted diet and demonstrate that eight weeks of chronic hypoxia (10%) is sufficient to induce RV hypertrophy and subsequent RV failure. Addition of a low copper diet to hypoxia did not have any further deleterious effects on right ventricular remodeling.

## Introduction

Pulmonary hypertension (PH) and subsequent right ventricular (RV) failure are associated with high morbidity and mortality. Prognosis is mainly determined by the ability of the RV to adapt to increased afterload, a key characteristic of PH. [1]–[5] Little is known about the mechanisms underlying the development of RV failure, and transition of RV hypertrophy to RV failure.

There are several animal models available to study RV failure. The most frequently used models are rodent-models, where exposure to either monocrotaline or hypoxia induces RV remodeling and failure. [6], [7] Chronic hypoxia induces both vasoconstriction and remodeling of the pulmonary vascular bed resulting in increased pulmonary pressure, leading to RV failure. [8], [9] This model was predominantly studied in rats, but recent studies demonstrated that mice exhibit a less severe pulmonary vascular remodeling when exposed to chronic hypoxia compared to rats. [9] In mice, chronic hypoxia induced RV hypertrophy and increased right ventricular systolic pressure (RVSP). [10]–[22] Although the effect of hypoxia on RV function remains largely unstudied, the few studies that did look at fractional shortening or RV cardiac output failed to show a decrease. [23], [24] However, one must note that this included only short-term exposure to hypoxia.

Other rodent models of pulmonary hypertension mostly involve multiple insults, including the combination of chronic hypoxia with VEGFR inhibition (SuHx) and monocrotalin treatment with pneumonectomy, to induce not only pulmonary vascular remodeling but also subsequent RV failure. [14], [25]–[27] In mice, SuHx is the only double-insult model previously described to induce RV failure [14].

Bogaard et al. showed that addition of a copper-depleted diet to pulmonary artery banding (PAB) in rats led to increased RV fibrosis and RV dilation as well as capillary rarefaction. [26] Low-copper diet interferes with HIF-1α protein stabilization, which is necessary for vascular endothelial growth factor (VEGF) expression, and therefore subsequently affects angiogenesis. [26], [28] Under hypoxic conditions, myofiber area increases two-fold, leading to a reduction in capillary density, however a small increase in capillary: fiber ratio is seen as a result of modest proliferation of capillaries secondary to RV hypertrophy. [29] It is known that maintaining cardiac function during hypertrophy is in part, angiogenesis-dependent, and that lack of VEGF expression contributes to the progression from adaptive cardiac hypertrophy to heart failure [30], [31].

In contrast, a copper-depleted diet prevented the development of severe experimental pulmonary hypertension in the rat model, which included VEGF receptor blockade with chronic hypoxia (SuHx model), by reducing obliteration of the small pulmonary vessels [32].

In recent years, the possibilities for genetic engineering in mice have significantly evolved, making the use of mice as an animal model highly attractive. It would therefore be beneficial to have murine models of right ventricular failure. We hypothesize that a low-copper diet added to chronic hypoxia in mice reinforces their individual effects and the combination of mild pulmonary vascular remodeling, with RV capillary rarefaction, induces RV failure. Therefore we propose to investigate the effect of a low copper diet in a murine model of chronic hypoxia on right ventricular function.

## Materials and Methods

### Animals

All animal handling and procedures were approved by the ethics committee of animal welfare at Maastricht University, and were in accordance with governmental guidelines.

A total of 48 C57BL/6 male mice were used at 6 weeks of age at the start of the experiment. Mice were housed at room temperature (20°C) and placed in a 12-hour light-dark cycle. Food and water were accessible ad libitum; food consumption was monitored and was similar for all four experimental groups, hence pair-feeding was deemed unnecessary throughout the experiment. Mice received either a copper depleted diet (Harlan Teklad, TD 80388) or a normal chow diet (Harlan Teklad LM-485).

For hypoxia, the animals were placed in a sealed chamber (n = 12 per experimental group); a fan circulated air within the chamber. O_2_ concentration was maintained at 10% by controlling the inflow rate of N_2_. Prior to keeping mice at 10% O_2_, they were allowed to adjust to their surroundings for 24 hours to minimize stress, and O_2_ levels were gradually decreased to 10% over an additional period of 72 hours. Littermates served as controls in the normoxia group and were kept in room air (21% O_2_); a fan ensured proper air circulation within the chamber. Chambers were unsealed for less than 15 minutes per day in order to replenish food, clean cages and check ventilators.

### Echocardiography

Mice were anaesthetized with isoflurane (mean 1.5% in oxygen), shaved and allowed to breathe spontaneously through a nasal cone. Non-invasive, echocardiographic parameters were measured using a digital cardiac ultrasound platform (Vevo 2100, VisualSonics); for cardiac parameters, the transducer was applied parasternally to the shaved chest wall, and measurements were performed as previously described [33].

### MRI

Mice were anaesthetized using isoflurane. Body temperature was monitored using a rectal temperature probe and mice were placed under a warm-water blanket. Additionally, respiratory rate was monitored continuously. MRI was performed using a 7 Tesla Bruker Biospec 70/30 USR (Bruker Biospin, Ettlingen, Germany).

The right ventricle was measured using an Intragate sequence, with a field of view of 25.6×25.6 mm, matrix was 256×256, TE 3 ms, TR, 60 ms, 7 slices. The cine calculated out of the Intragate sequence consists of 15 frames. All images were analyzed in OsiriX (Dicom viewer, version 3.5, Pixmeo, Geneva, Switzerland). The RV end-diastolic and RV end-systolic volumes were analysed using multi-slice short axis cine-images of the complete right ventricle. RV ejection fraction was calculated as (RVEDV-RVESV)/RVEDV *100.

### Assessment of RV Hypertrophy

The heart was dissected and both atria, the aorta, and the pulmonary trunk were removed. The right ventricle was separated from the left ventricle and the ventricular septum. Right ventricular hypertrophy was expressed using the Fulton index; the ratio of the weight of the right ventricular wall to the left ventricular wall and ventricular septum (RV/LV+S).

### Histology, Immunohistochemistry and Immunofluorescence

Heart- and lung specimens were fixed and cleared of blood by perfusion with 3.8% paraformaldehyde and subsequently embedded in paraffin. Tissue sections (4 µm) were stained with hematoxylin and eosin (H&E) for routine histological anaylsis, or with picro-Sirius red for visualization of collagen deposition. Additionally, FITC-labeled wheat-germ-agglutinin (WGA) was used to visualize and quantify cross-sectional cell area; capillary density was visualized and quantified using a Griffonia simplicifolia agglutin-I (GS-I) lectin stain.

Slides were visualized and imaged using a Zeiss Axioskop 2Plus with an AxioCamHRc.

Fulton-index was used as an index for RV hypertrophy and calculated as the ratio of the RV free wall weight over the septum plus left ventricular free wall weight.

### Real-time PCR

Primers were designed to detect transcripts for *nppa* (NM_008725, 5′-TCTTCCTCGTCTTGGCCTTT, 5′-CCAGGTGGTCTAGCAGGTTC), *nppb* (NM_008726, 5′-TGGGAGGTCACTCCTATCCT, 5′-GGCCATTTCCTCCGACTTT), *acta1* (NM_009606, 5′-CCGGGAGAAGATGACTCAAA, 5′-GTAGTACGGCC GGAAGCATA), *myh7* (NM_080728, 5′-CGGACCTTGGAAGACCAGAT, 5′-GACAGC TCCCCATTCTCTGT) and *rcan1.4* (NM_019466, 5′-GCTTGACTGAGAGAGCGAGTC, 5′-CCACACAAGCAATCAGGGAGC).

RNA was isolated from tissue using TRIzol reagent (Invitrogen) RNA (1 µg) from right ventricular mouse heart tissue was reverse-transcribed using Superscript II reverse transcriptase (Invitrogen). Real-time PCR was performed on a BioRad iCycler (Biorad) using SYBR Green. Transcript quantities were compared using the relative Ct method, which normalizes the amount of target to the amount of endogenous control (L7) relative to the control sample, and are given by 2−ΔΔCt.

### Statistical Analysis

The results are presented as mean ± standard error of the mean (s.e.m.). Statistical analyses were performed using Prism software (GraphPad Software), and consisted of ANOVA followed by Tukey’s post-test when group differences were detected at the 5% significance level, or Student’s t-test when comparing two groups. Differences were considered significant when P<0.05.

## Results

### Chronic Hypoxia Induces RV Hypertrophy

**Figure 1:**
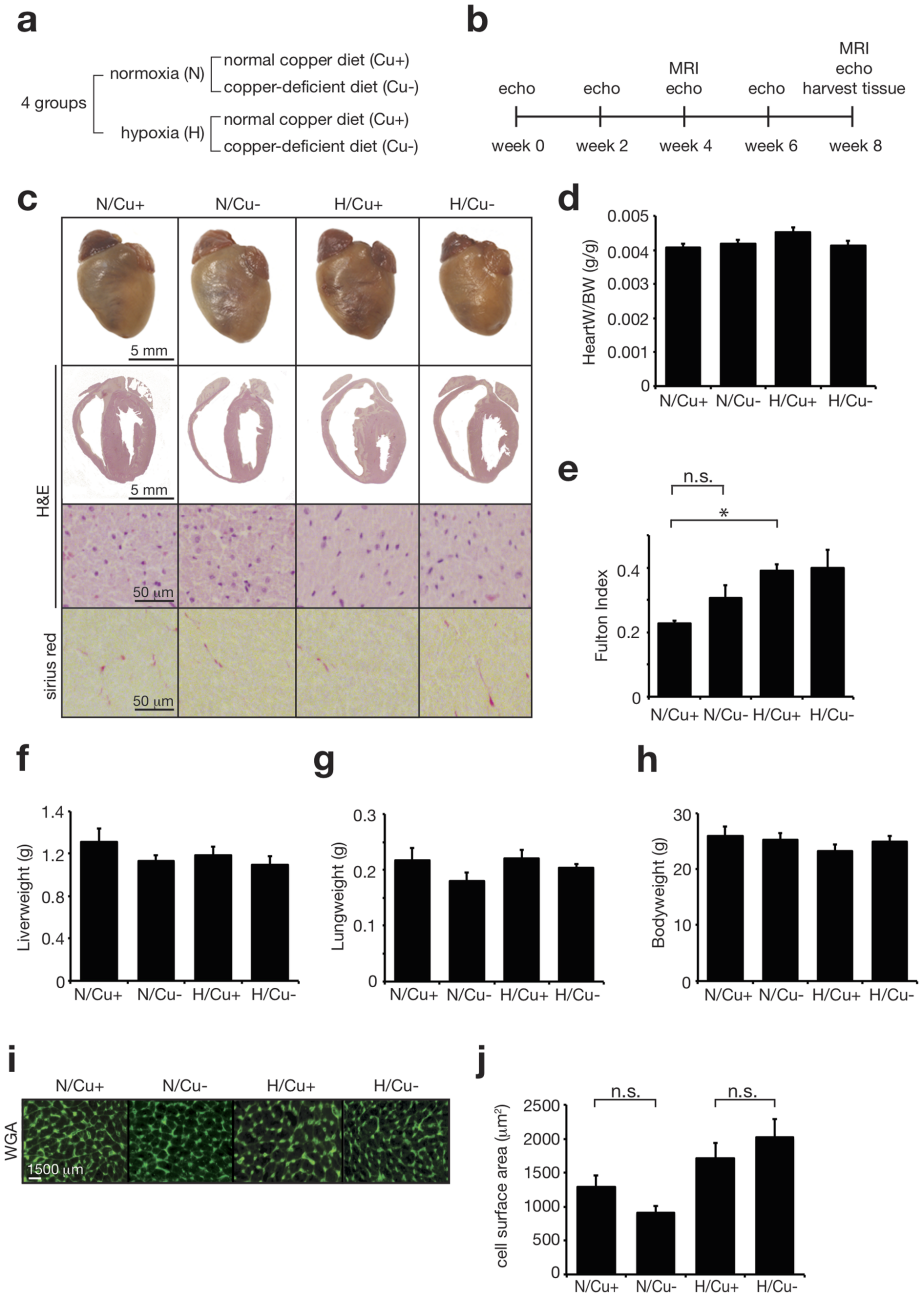
Chronic hypoxia leads to an increase in Fulton index. (a) Experimental groups. (b) Study design. (c) Representative images of whole hearts (upper panels), haematoxylin & eosin (H&E) stained 4-chamber paraffin sections (second panels), high magnification H&E stained free right ventricular wall histological sections (third panels), high magnification sirius red stained free right ventricular wall sections (lower panels. The following groups are compared: normoxia on normal chow diet (N/Cu+), normoxia on copper depleted diet (N/Cu-), hypoxia on normal chow diet (H/Cu+) and hypoxia on copper depleted diet (H/Cu-). (d) Heartweight corrected for bodyweight in grams per gram. (n = 9) *P<0.05. (e) Fulton index calculated as ratio of RV free wall weight over septum plus LV free wall weight. (f) Liverweight in grams. (g) Lungweight in grams. (h) Bodyweight in grams. (i) Representative images of wheat germ agglutinin (WGA) stained sections. (j) Quantification of average cell surface area using WGA stained sections. (n≥610) *P<0.05 (mean ± s.e.m.).

In order to study the effect of a low copper diet on right ventricular function in a murine model of chronic hypoxia we used four different wildtype mice groups subjected to different conditions as depicted in Figure 1a. The animals were followed for 8 weeks, during which cardiac function was analyzed every two weeks by echocardiography and complemented with MRI analysis at week 4 and 8 (Figure 1b). Pictures of whole hearts and HE stains, after 8 weeks of hypoxia, showed normal tissue morphology and absence of inflammatory cell infiltration for all groups. Additionally, there was no significant increase in fibrosis, assessed using Sirius Red staining, in hypoxia groups when compared to normoxia (Figure 1c). Moreover, the difference in mean heart weight corrected for body weight between normoxia/Cu+ (4.1±0.1 g/g), normoxia/Cu- (4.2±0.1 g/g), hypoxia/Cu+ (4.5±0.1 g/g), hypoxia/Cu- (4.1±0.1 g/g) did not differ (Figure 1d). Right ventricular weight, expressed using Fulton index (RV: (LV+septum)), was increased in hypoxia/Cu+ mice (0.39±0.02) compared to normoxia/Cu+ mice (0.23±0.01; p = 0.033; Figure 1e), indicating that hypoxia was sufficient to induce hypertrophic growth of the right ventricle. There was no further increase in Fulton index after adding a copper deficient diet to hypoxia treatment (hypoxia/Cu- 0.40±0.0.06, p = 0.988), indicating no additional effect of a copper deficient diet on RV hypertrophy. Cell surface area demonstrated a trend towards an increase when comparing hypoxia to normoxia groups (Figure 1i, j). Mice showed no clinical signs of overt heart failure, e.g. no peripheral edema. Liver- and lung weights were normal and showed no significant differences between groups (p = 0.337, p = 0.223 respectively) (Figure 1f, g). This data correlates with the observed heart weights in Figure 1d. Furthermore, mice showed no drop in body weight after the introduction of hypoxia (normoxia/Cu+: 26.0±1.6 g, hypoxia/Cu+: 23.3±1.2 g, p = 0.528; Figure 1h).

### Combination of Low-copper Diet with Hypoxia does not Exacerbate RV Failure

**Figure 2:**
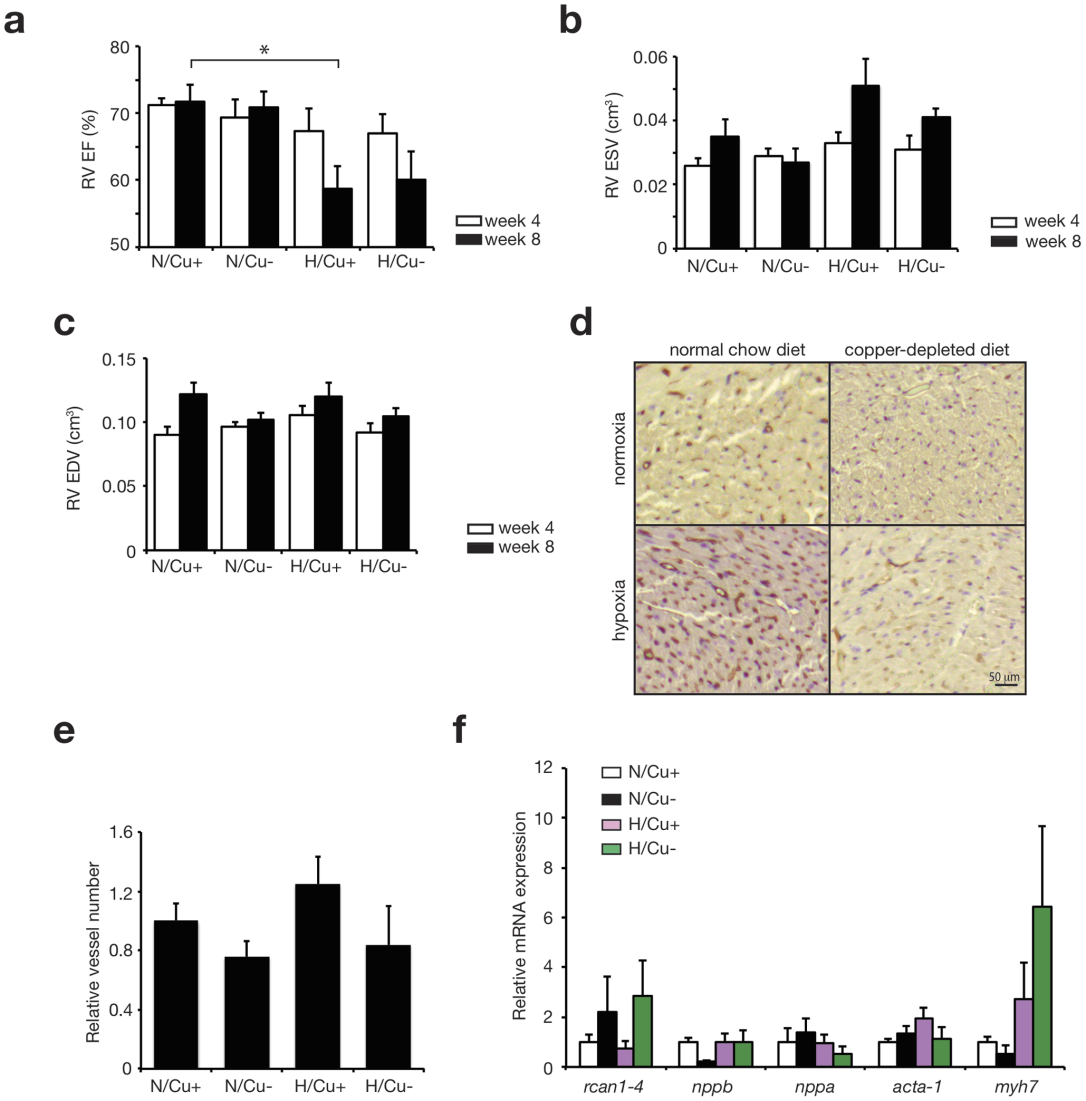
Chronic hypoxia induces a decrease in right ventricular ejection fraction. Right ventricular ejection fraction (EF) measured by MRI at 4 and 8 weeks. The following groups are compared: normoxia on normal chow diet (N/Cu+), normoxia on copper depleted diet (N/Cu), hypoxia on normal chow diet (H/Cu+) and hypoxia on copper depleted diet (H/Cu-). (b) Right ventricular end systolic volumes in cm^3^ and (c) right ventricular end diastolic volumes in cm^3^. (n = 6) (d) Griffonia simplicifolia 1 (GS-1)-stain on representative right ventricular free wall paraffin sections. (e) Quantification of GS-1 stain, expressed as relative vessel number. (f) Real-time PCR analysis of transcript abundance for cardiac stress marker genes in right ventricles at 8 weeks. (n = 3) *P<0.05 (mean ± s.e.m.).

RV ejection fraction was decreased after 8 weeks in hypoxia/Cu+ mice (58.8±3.3) compared to normoxia/Cu+ mice (71.7±2.6%, p = 0.05), demonstrating that hypoxia lead to RV failure (Figure 2a). However combination of low-copper diet with hypoxia did not have an additional detrimental effect with regard to RV failure. Interestingly, the RV end-systolic and end-diastolic volumes remained unchanged between all four groups at both 4 and 8 weeks (Figure 2b, c).

### Hypoxia nor Low-copper Diet Affected Capillary Density

Capillary density was determined by a GS-1 stain, showing no differences between hypoxia/Cu-, hypoxia/Cu+, nor normoxia/Cu- and normoxia/Cu+ mice (Figure 2d, e).

### Stress Markers

Real-time PCR was performed to determine the expression levels of cardiac stress markers in the right ventricle (Figure 2f). There were no significant changes in cardiac stress marker expression when comparing all experimental conditions. We have also assessed Calcineurin-NFAT signaling activity by determining rcan1.4 expression at the mRNA level. Although there seems to be a trend for increased expression under a copper-deficient diet, this effect was not statistical significant. Furthermore, no differences were observed when comparing all 4 groups.

## Discussion

This study demonstrates that exposing mice to 8 weeks of hypoxia induces RV failure. The addition of a copper depleted diet to hypoxia did not have an additional deleterious effect on RV function. Furthermore copper depletion alone did not have an effect on RV hypertrophy or RV function.

Dempsey at al. showed that susceptibility to chronic hypoxia varies between species and murine susceptibility strongly depends on genetic background. [9], [34] Response to hypoxia was also significantly affected by the age of the animal; ‘infant’ rats of 8 days old with maturing lungs were more susceptible to a hypoxic trigger compared to older, adult animals. [8] [35] We used C57BL/6 mice and started the experiment at an adult age with 6 weeks-old mice, however it is possible that using a different strain of mice and/or starting the experiment at a younger age elicits a different response to hypoxia. Vascular remodeling after exposure to hypoxia was less significant in mice compared to rats. [36] Even when VEGF blocker was added to hypoxia in mice this did not lead to the typical plexiform lesions described in rats. [14] Several studies have previously shown that 3 weeks of hypoxia (10% O_2_) in mice leads to an increase in RV hypertrophy, and our data indicate that prolonged hypoxia exposure also results in a larger increase in Fulton index. [14], [24], [37] Although RV dilation was shown previously, our model demonstrates that chronic hypoxia for 8 weeks induces RV failure.

Our study demonstrated that copper depleted diet combined with exposure to hypoxia did not influence RV hypertrophy. During sustained pressure overload, cardiac copper and VEGF levels decreased, coinciding with a suppression of myocardial angiogenesis. [28] Previous studies demonstrated that copper supplementation reverses hypertrophy by copper stimulation of VEGF production through activation of HIF1a. [38], [39] This is in accordance with the fact that copper depleted diet interfered with angiogenesis, which is needed to sustain RV hypertrophy. Additionally, it has been shown that copper depletion itself, in the absence of other forms of stress on the ventricle, can induce ventricular hypertrophy, mainly through an increase in mitochondrial volume density as well as in increase in mitochondria. [40] Furthermore, lack of cardiac copper promoted cardiac electrical conduction abnormalities, including ventricular fibrillation and heart block, which could be abrogated by adequate copper supplementation. [40]–[42] We, however, did not observe any dropout in our animal groups on copper depleted diet, suggesting that there were no lethal cardiac conduction disturbances. Perhaps prolonging the study duration beyond 8 weeks would increase the risk and induction of arrhythmias, although it is known that mice and rats are more resistant to the development of cardiac conduction abnormalities than other species.

Copper is needed in the development of severe pulmonary hypertension and the formation of angioproliferative lesions in the SuHx model. [32] Although these lesions resemble plexiform lesions typical for pulmonary artery hypertension, patients with pulmonary hypertension due to left heart disease and/or lung disease, which represent the majority of PH patients do not present with plexiform lesions. [43] Furthermore, copper depletion did not influence the degree of pulmonary artery media thickness in the SuHx rat model, or influence pulmonary pressures, RV hypertrophy or media thickness. We did not see an influence of copper in the development of RV hypertrophy or the development of RV failure in our hypoxia mice model.

During hypertrophy, the ventricle reaches a critical point where the amount of capillaries can no longer sustain the amount of ventricular tissue and the ventricle will rapidly start to fail.

We are aware that our results may be the consequence of the timeframe of our experimental setup (8 weeks under hypoxic conditions) and it is likely that increasing the period of hypoxia will strengthen the effect of adding copper depleted diet to chronic hypoxia. Nevertheless, eight weeks of hypoxia was sufficient to induce RV failure in mice.

The fact that cardiac stress markers are not increased in hypoxia could be explained by differences in stress response between the right and left ventricles. Accordingly, it is known that the right ventricle reacts differently to stress by activating different mechanisms and pathways. [44], [45] Therefore, it is reasonable to think that under chronic stress conditions, different markers are induced in the right ventricle compared to the left ventricle. On the other hand, the degree of RV hypertrophy and failure caused by 8 weeks of exposure to 10% hypoxia may be insufficient to induce increased expression levels of the tested cardiac stress markers. A recent study has implicated NFAT activation as a requirement for chronic hypoxia-induced pulmonary hypertension. [46] Since activation of NFAT signaling is a direct modulator of the left ventricle response to cardiac stress [47] leading to left ventricle hypertrophic growth and eventually heart failure, we investigated whether chronic hypoxia leads to RV hypertrophy through direct activation of NFAT transcription factors. RCAN1.4 gene expression, a target gene of the calcineurin/NFAT pathway, responsive to NFAT activation, did not significantly change between all groups. Although a trend for increased RCAN1.4 expression under a low-copper diet was observed, suggesting that copper-deficiency induced cardiac stress may lead to calcineurin/NFAT signaling activation, more investigation is needed to clarify this matter.

In conclusion, we demonstrate that prolonged exposure to hypoxia induces RV failure. Prolonged exposure to hypoxia leads to more pronounced RV hypertrophy, compared to short-term exposure. [10]–[22] Finally, dietary copper deficiency did not have any further deleterious effects on RV function.
